# Maternal gut microbiota transmission and early-life colonization patterns influence infant CMPA risk

**DOI:** 10.1128/spectrum.01162-25

**Published:** 2025-10-20

**Authors:** Lai Zhang, Xiaomeng Ge, Peiliang Shen, Yirong Zhou, Songnian Hu, Huisong Xu

**Affiliations:** 1 Hangzhou Linping District Maternal and Child Health Care Hospital 669374 , Hangzhou, China; 2 Microbial Resources and Big Data Center, Institute of Microbiology, Chinese Academy of Sciences 85387, Beijing, China; 3 State Key Laboratory of Microbial Resources, Institute of Microbiology, Chinese Academy of Sciences 85387, Beijing, China; 4 University of Chinese Academy of Sciences, Beijing, China; USDA-ARS Arkansas Children's Nutrition Center, Little Rock, Arkansas, USA

**Keywords:** maternal-infant microbiota, food allergy, CMPA, feeding patterns, *Bifidobacterium*

## Abstract

The establishment of gut microbiota in early life represents a critical window for immune system development and the prevention of cow’s milk protein allergy (CMPA). While maternal influence on infant gut colonization is well-documented, the specific relationship between maternal-infant microbiota patterns and CMPA development remains poorly understood. In this study, we analyzed 106 fecal samples from maternal-infant pairs, including 16 complete triads (maternal, newborn, and 3-month infant samples), using 16S rRNA sequencing to identify key bacterial markers associated with CMPA risk. Our findings revealed distinct temporal colonization patterns, with initial *Staphylococcus* dominance in newborns shifting to *Bifidobacterium* predominance by three months, a trend particularly pronounced in infants with CMPA. Breastfeeding emerged as a significant factor influencing microbial development, with exclusively breastfed infants showing a lower risk of CMPA. Phylogenetic analysis confirmed the vertical transmission of *Staphylococcus*, *Bifidobacterium* (such as *Bifidobacterium longum* and *Bifidobacterium breve*), *Blautia*, and *Enterococcus* between mothers and infants. Furthermore, functional pathway analysis highlighted the role of amino acid metabolism in immune system development, suggesting potential mechanisms through which early microbiota may influence CMPA risk. These findings provide new insights into the interplay between maternal-infant microbiota transmission, feeding practices, and CMPA development, offering potential avenues for early intervention and prevention strategies.

**IMPORTANCE:**

The establishment of intestinal flora in early life is essential for the health and development of living organisms. At birth, the newborn has already established an initial gut microbiota with distinct characteristics. The link between the composition of the gut microbiota and immune-related symptoms such as allergies is a hot topic, and it starts to show up early in life. It is helpful to study the characteristics and developing rules of early life intestinal flora by interpreting the characteristics of early life flora, the relationship with maternal flora, the development law with age, and the correlation with feeding patterns and so on. In this study, we presented and compared the gut microbiota of newborns, 3-month-old infants, and their mothers to provide a preliminary understanding of the establishment of early life microbiota and its correlation with allergies.

## INTRODUCTION

The prevalence of allergic diseases has been increasing in many countries ((1)). In the United States, 3.9% of US children are reported as having food allergy, with an 18% increase in prevalence between 1997 and 2007 (2). In a telephone survey involving 8,825 European children, cow’s milk was reported as the most common food allergen (38.5%) (3). Infant cow’s milk protein allergy (CMPA), a significant public health issue, has an estimated incidence of 2–5% in infants at 1 year of age in resource-rich countries such as the United States, Germany, and Spain (4). It was reported that eggs and milk are the most common food allergens among children in Shanghai, which is close to the sampling site of this study (Hangzhou in Zhejiang province), and both areas are urban environments (5, 6). CMPA can significantly impact a child’s quality of life and impose a financial burden on families (7).

CMPA can be mediated by IgE-dependent mechanisms, non-IgE pathways, or a combination of both, resulting in a broad spectrum of clinical manifestations ranging from immediate allergic reactions to delayed gastrointestinal symptoms (8). While the exact pathogenesis of CMPA remains unclear, current research points to a complex interplay of genetic susceptibility, environmental factors, and alterations in the gut microbiome (9, 10). Emerging evidence suggests that gut microbiota composition and early-life microbial colonization may play a critical role in the development of CMPA (11). Gastrointestinal symptoms such as vomiting, constipation, and diarrhea, which are common in CMPA, further highlight the importance of gut health in the manifestation of this condition (12).

Early childhood is a critical stage for the foundation and development of the microbiome, and microbial colonization early in life strongly influences human health and disease throughout life (13). The development of a healthy gut microbiota and immune system early in life occurs primarily through exposure to maternal microbes through vaginal/natural childbirth and breast milk (14). Early-life antibiotic exposures, cesarean section, and formula feeding could disrupt microbiome establishment and adversely affect health later in life (15, 16). Feeding practices, such as breastfeeding or formula feeding, have been shown to significantly influence the infant gut microbiome (17) and potentially impact CMPA development (18). For example, a study has demonstrated that specific microbial strains, such as *Bifidobacterium breve*, can modulate the immune system toward anti-allergic processes during early microbiota colonization (19). This suggests that maternal microbial transfer may play a crucial role in shaping the infant’s immune responses and reducing the risk of allergic conditions.

Despite growing evidence linking gut microbiota to CMPA, the specific microbial signatures associated with the onset and progression of CMPA remain poorly understood. To better understand the interplay between intestinal flora, feeding practices, and CMPA development, we designed a study to investigate the origin, compositional characteristics, and dynamic evolution of microflora in early life. Previous studies have shown that by three months of age, infants have initially established a relatively stable intestinal microbiota structure in the earliest period of life, and the further development generally commences around 6 months of age (20). In addition, some research has suggested that the lower microbiota richness in infants at three months is a risk factor for food allergies in infants at 1 year old (21). To avoid the influence of factors such as the introduction of complementary foods, we take 3 months as the time node of this study. Our study aims to address these gaps by longitudinally tracking the gut microbiota of mothers, newborns, and infants at 3 months of age. We seek to identify microbial markers that may predict CMPA risk. Specifically, we examine intestinal microbial characteristics across different age groups and compare allergic and healthy cohorts. These findings could contribute to CMPA risk assessment and inform future prevention strategies.

## RESULTS

### Sample composition based on maternal-infant allergy status

Infant allergic status was determined based on the presence of symptoms after the first 3 months of life, with symptom presentation remaining consistent throughout this period. A two-letter notation system (e.g., “Y_N”) was employed to represent the allergic phenotype of each maternal-infant pair (Table 1). The first letter indicates maternal allergy history and the second denotes infant allergic status. Allergic-positive samples include both CMPA and eczema cases. While some maternal samples were unavailable for retrospective analysis and several infants were lost to follow-up at 3 months, certain patterns emerged. Among infants born to allergic mothers, the distribution between those who developed allergies (Y_Y) and those who did not (Y_N) showed no clear pattern. In contrast, infants of non-allergic mothers predominantly remained symptom-free, with only one case developing CMPA symptoms (N_Y). More detailed sample information was provided in Table S1.

**TABLE 1:** Sample sizes of maternal allergy history and infant allergic phenotypes at birth (m0) and 3 months (m3)

Phenotypes	Maternal	Infant_m0	Infant_m3
Y_Y	13	15	10
Y_N	13	19	6
N_N	8	15	4
N_Y	1	1	1

### Changes in gut microbiota diversity and composition from mothers to infants over 3 months

**Fig 1:**
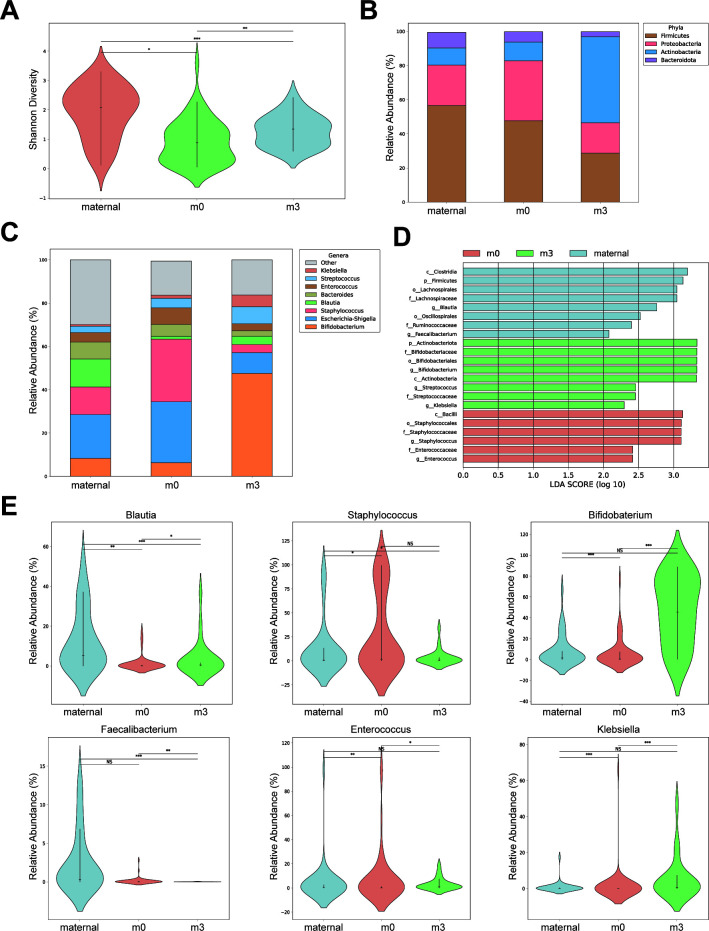
Temporal changes in gut microbiota composition across maternal and infant groups. (**A**) Shannon diversity index comparison between maternal (*n* = 35), newborn (m0, *n* = 50), and 3-month infant (m3, *n* = 21) samples. (**B**) Relative abundance of bacterial phyla across groups. (**C**) Most abundant bacterial genera in maternal and infant samples. (**D**) Linear discriminant analysis (LDA) scores (log10) for differentially abundant taxa identified by LEfSe between maternal, m0, and m3 groups. (**E**) Temporal abundance patterns of six key bacterial markers identified by LEfSe analysis. Data are presented as mean ± SD. Wilcoxon rank-sum test was used for all statistical comparisons, *P < 0.05.

16S sequencing was performed on fecal samples from mothers and infants to investigate differences in gut microbiota and explore potential associations between maternal-infant allergy transmission. Notably, infant gut microbiota diversity increased considerably after 3 months (m3) compared to birth (m0) (Fig. 1A). Despite the lowest diversity, newborn infant gut bacteria showed high congruence with maternal microbiota at the phylum level, suggesting retention of core bacteria during labor and delivery. At 3 months, a substantial increase in the proportion of Actinobacteria phylum was observed, possibly due to factors like feeding practices and environmental changes from utero to home (Fig. 1B). At the genus level, newborn infants predominantly inherited *Escherichia-Shigella* and *Staphylococcus* (Fig. 1C). *Escherichia-Shigella* is a common intestinal bacterium, while some *Staphylococcus* is frequently found in the vaginal microbiome. The large increase in Actinobacteria in 3-month infant samples was primarily attributed to the genus *Bifidobacterium. Bifidobacterium* is not only a common intestinal bacterium but also the genus containing many of the most common and commercially available probiotic strains.

### Identification of key bacterial markers across maternal and infant groups

Linear discriminant analysis effect size (LEfSe) analysis was performed to identify differential bacterial markers between maternal and infant gut microbiota. *Staphylococcus* emerged as a dominant marker in the newborn (m0) group, while *Bifidobacterium* characterized the 3-month (m3) group. In the maternal group, the genus *Blautia* exhibited the most significant differential abundance (Fig. 1D; Table 2). Further analysis of six key bacterial markers identified by LEfSe across all groups revealed distinct temporal patterns (Fig. 1E). *Blautia*, while showing a slight increase from m0 to m3, maintained its dominance in the maternal group, suggesting an age-dependent accumulation. *Faecalibacterium*, another maternal group marker, showed limited initial colonization in m0 infants and further decreased by m3, possibly reflecting the greater oxygen tolerance and transmission potential of *Blautia* compared to the strict anaerobe *Faecalibacterium* (22). *Staphylococcus* and *Enterococcus*, both common facultative anaerobes in the vaginal microbiota, emerged as characteristic markers of the m0 group, reflecting their role as pioneer colonizers. *Bifidobacterium* showed marked proliferation to become a dominant marker in the m3 group, with *Klebsiella* exhibiting a similar developmental pattern.

**TABLE 2:** Identified genus-level markers and their LDA scores in maternal-infant (maternal, *n* = 35; m0, *n* = 50; m3, *n* = 21) groups

Taxonomies	Groups	*P* value	LDA scores
d__Bacteria.p__Firmicutes.c__Bacilli.o__Staphylococcales.f__Staphylococcaceae.g__*Staphylococcus*	m0	5.99E−03	3.1070
d__Bacteria.p__Actinobacteriota.c__Actinobacteria.o__Bifidobacteriales.f__Bifidobacteriaceae.g__*Bifidobacterium*	m3	6.42E−07	3.3306
d__Bacteria.p__Firmicutes.c__Clostridia.o__Lachnospirales.f__Lachnospiraceae.g__*Blautia*	Maternal	2.08E−05	2.7606
d__Bacteria.p__Firmicutes.c__Bacilli.o__Lactobacillales.f__Enterococcaceae.g__*Enterococcus*	m0	2.67E−02	2.4147
d__Bacteria.p__Proteobacteria.c__Gammaproteobacteria.o__Enterobacterales.f__Enterobacteriaceae.g__*Klebsiella*	m3	1.85E−07	2.2951
d__Bacteria.p__Firmicutes.c__Clostridia.o__Oscillospirales.f__Ruminococcaceae.g__*Faecalibacterium*	Maternal	1.47E−04	2.0776

### Distinct microbial community patterns between allergic and non-allergic groups

**Fig 2:**
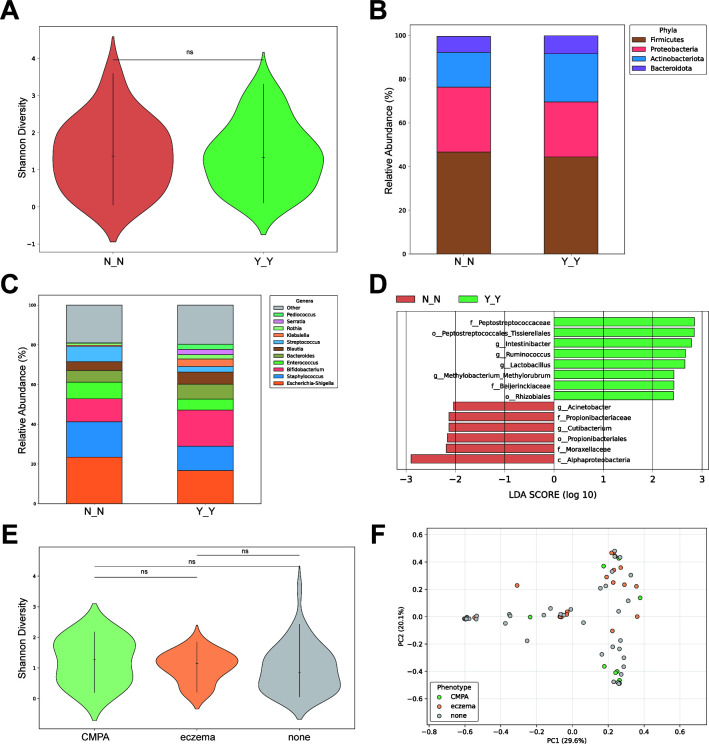
Comparative analysis of microbial communities between allergic (Y_Y) and non-allergic (N_N) groups. (**A**) Shannon diversity index comparison between allergic (Y_Y, *n* = 38) and non-allergic (N_N, *n* = 27) groups. (**B**) Relative abundance of bacterial phyla across groups. (**C**) Most abundant bacterial genera in allergic and non-allergic samples. (**D**) Linear discriminant analysis (LDA) scores (log10) for differentially abundant taxa identified by LEfSe between allergic and non-allergic groups. (**E**) Shannon diversity index comparison among CMPA (*n* = 10), eczema (*n* = 17), and non-allergic (none, *n* = 44) groups in infant samples. (**F**) Principal coordinate analysis (PCoA) based on Bray-Curtis distances of infant samples (*n* = 71). Data are presented as mean ± SD. Wilcoxon rank-sum test was used for all statistical comparisons, *P < 0.05.

To explore microbial differences associated with allergic phenotypes, we compared N_N and Y_Y samples across all timepoints (maternal, m0, and m3). In contrast to the diversity between maternal-infant samples, there was no significant difference in microbial diversity between the allergic and non-allergic groups (Fig. 2A). At the phylum level, the allergic group possessed somewhat higher abundance of Actinobacteria and lower abundance of Proteobacteria (Fig. 2B). The abundance of the top-ranked genera in the allergic group showed a more even distribution compared to the non-allergic group (Fig. 2C). LEfSe analysis revealed that none of the identified microbial markers of allergy were among the highly abundant genera (Fig. 2C), indicating that allergic status influences microbial composition through subtle modulations of low-abundance taxa rather than major shifts in dominant bacteria (Fig. 2C and D; Table 3).

**TABLE 3:** Identified genus-level markers and their LDA scores between allergic (Y_Y, *n* = 38) and non-allergic (N_N, *n* = 27) groups

Taxonomies	Groups	*P* value	LDA scores
d__Bacteria.p__Firmicutes.c__Clostridia.o__Peptostreptococcales_Tissierellales.f__Peptostreptococcaceae.g__*Intestinibacter*	Y_Y	4.61E−03	2.7855
d__Bacteria.p__Firmicutes.c__Clostridia.o__Oscillospirales.f__Ruminococcaceae.g__*Ruminococcus*	Y_Y	1.50E−02	2.6593
d__Bacteria.p__Firmicutes.c__Bacilli.o__Lactobacillales.f__Lactobacillaceae.g__*Lactobacillus*	Y_Y	2.38E−02	2.6503
d__Bacteria.p__Proteobacteria.c__Alphaproteobacteria.o__Rhizobiales.f__Beijerinckiaceae.g__*Methylobacterium_Methylorubrum*	Y_Y	1.56E−02	2.4280
d__Bacteria.p__Actinobacteriota.c__Actinobacteria.o__Propionibacteriales.f__Propionibacteriaceae.g__*Cutibacterium*	N_N	3.67E−04	2.1294
d__Bacteria.p__Proteobacteria.c__Gammaproteobacteria.o__Pseudomonadales.f__Moraxellaceae.g__*Acinetobacter*	N_N	2.16E−02	2.0375

### Microbial diversity and compositional differences between CMPA and eczema

To further exclude maternal influence and investigate differences between CMPA and eczema samples, we conducted additional analyses focusing on infant samples. Although no significant differences in Shannon diversity were observed among eczema, CMPA, and non-allergic samples, both eczema and CMPA groups displayed slightly higher median diversity compared to non-allergic infants, suggesting a potential consistency between the two allergic phenotypes (Fig. 2E). Principal coordinate analysis (PCoA) further revealed partial separation between CMPA and eczema samples, although the separation trend was not strongly pronounced, likely due to the limited sample size (Fig. 2F).

### Temporal patterns of microbiota function in relation to allergy phenotypes

**Fig 3:**
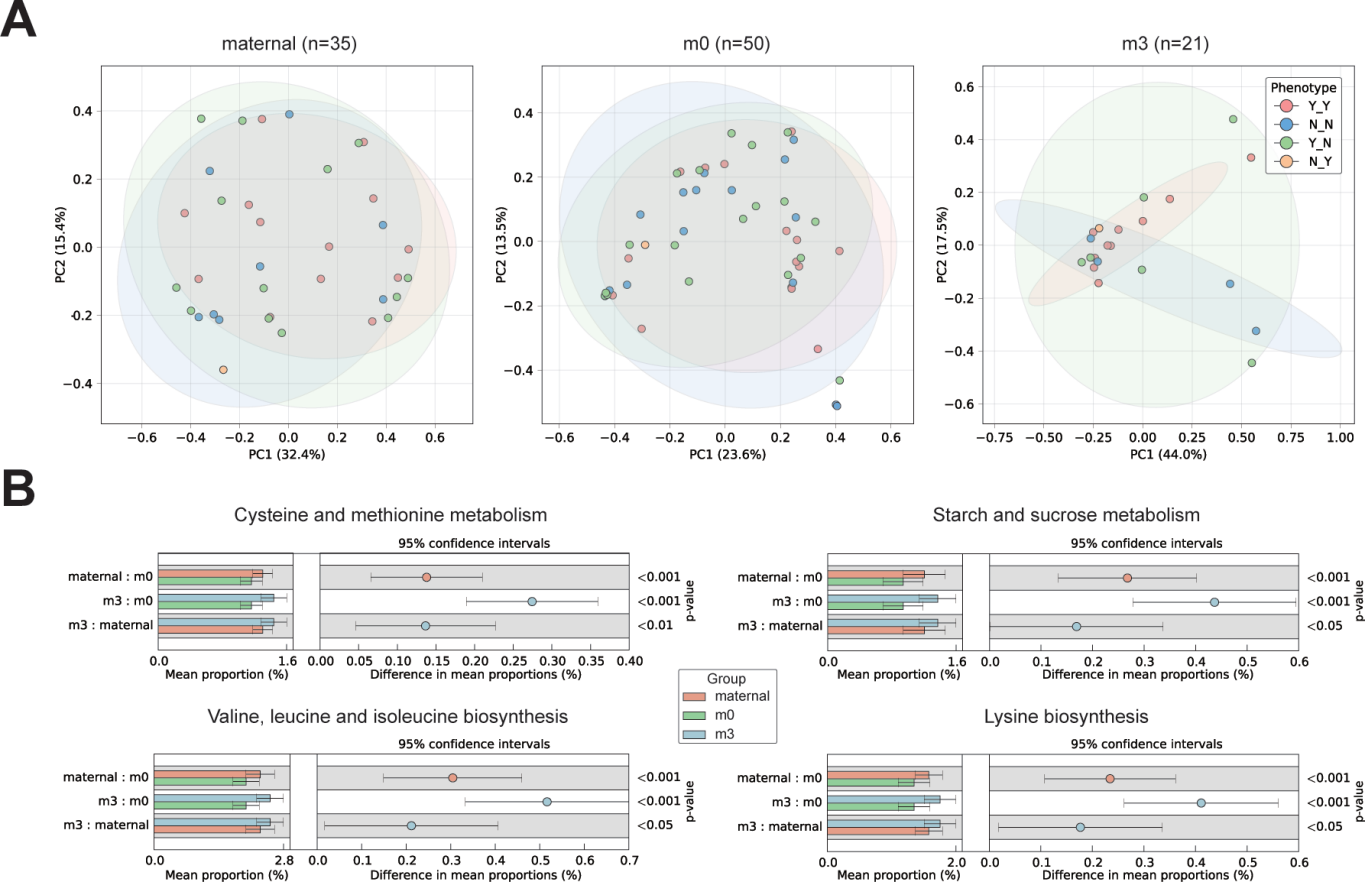
Functional analysis of gut microbiota in relation to allergy phenotypes. (**A**) PCoA based on Bray-Curtis distances showing microbial community relationships between maternal (*n* = 35), m0 (*n* = 50), and m3 (*n* = 21) samples across different allergic phenotypes. (**B**) Differential functional pathways identified by PICRUSt2 and STAMP analysis, highlighting significant metabolic differences between groups with post hoc plot. Pairwise comparisons were performed using the Kruskal-Wallis *H*-test, *P < 0.05.

PCoA of beta diversity showed substantial overlap between maternal and newborn (m0) microbial communities across allergic phenotypes, while 3-month (m3) samples formed distinct clusters, indicating substantial postnatal restructuring of the infant gut microbiome. Further, the unique microbiota distribution patterns of the different samples in the m3 group also suggest that while vertical transmission establishes the initial microbial foundation, early environmental exposures and feeding practices play crucial roles in shaping the developing infant gut microbiome and subsequent CMPA risk (Fig. 3A). Functional prediction using Phylogenetic Investigation of Communities by Reconstruction of Unobserved States 2 (PICRUSt2) identified four significant metabolic pathways, predominantly enriched in m3 samples. These included three amino acid metabolism pathways (Cysteine and methionine metabolism, *P* = 2.18E−9, Valine, leucine and isoleucine biosynthesis, *P* = 1.90E−8, and Lysine biosynthesis, *P* = 3.23E−8) and one carbohydrate metabolism pathway (Starch and sucrose metabolism, *P* = 1.64E−8).

### Maternal-infant microbiota transmission patterns in relation to infant allergy development

To investigate maternal-infant microbiota transmission patterns, we classified samples from 16 maternal-infant pairs into enterotypes based on the predominant genus (Fig. 1C). Three distinct enterotypes emerged: maternal samples showed *Blautia* dominance, newborn (m0) samples showed *Staphylococcus* dominance, and 3-month (m3) samples showed *Bifidobacterium* dominance. Notably, all allergic infants (8/16) exhibited *Bifidobacterium*-dominant profiles at 3 months, regardless of maternal status. Among breastfed infants (5/16), 80% of mothers (4/5) had allergies, yet all of these infants remained allergy-free. In contrast, artificially fed infants (2/16) uniformly developed allergy and showed consistent *Bifidobacterium* dominance at 3 months.

### Phylogenetic evidence for maternal-infant bacterial vertical transmission

**Fig 4:**
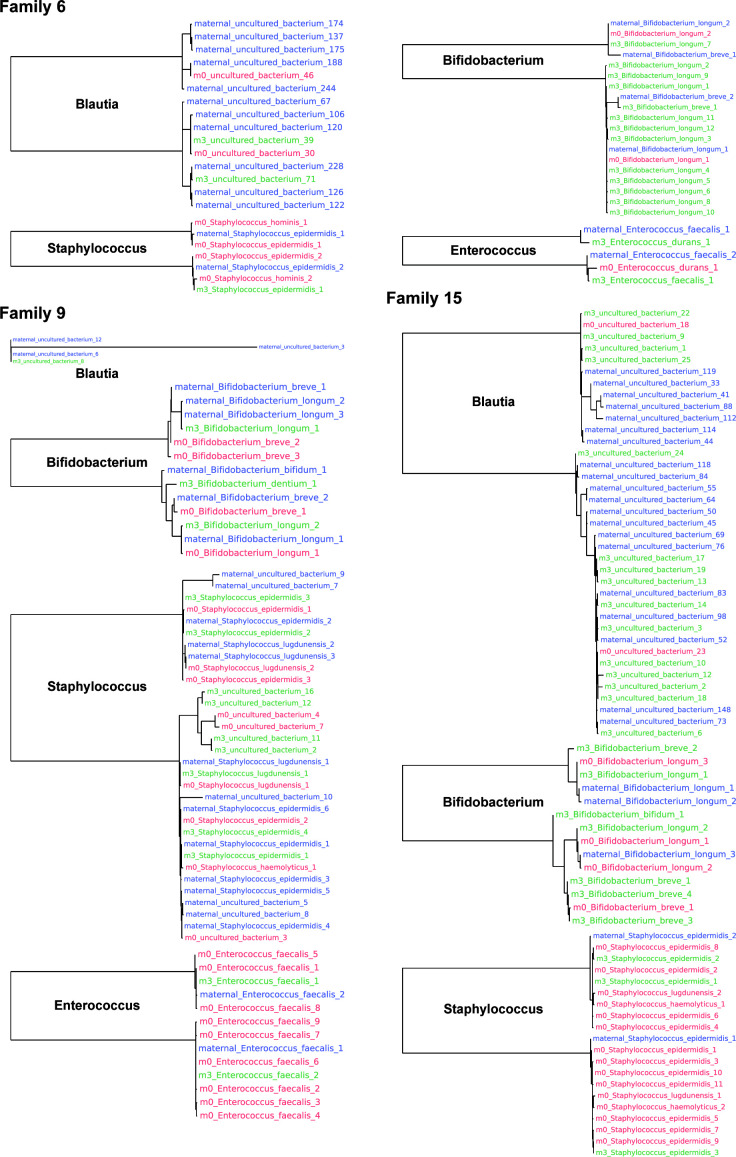
Phylogenetic analysis of maternal-infant bacterial vertical transmission in three representative families. Phylogenetic trees showing bacterial strain relationships in Families 6, 9, and 15, which cover different enterotype patterns (Family 6, *Blautia*-Mix-*Bifidobacterium*; Family 9, Mix-Mix-Mix; and Family 15, *Blautia-Staphylococcus-Bifidobacterium*), feeding methods (Family 6, Artificial; Family 9, Mixed; and Family 15, Breast), and maternal-infant allergic phenotypes (Family 6, Y_Y; Family 9, N_N; and Family 15, Y_N). ASVs of the same species are distinguished by numerical suffixes. Leaf colors represent sample origin (purple, maternal; red, m0; and green, M3). Phylogenetic trees of a genus within a family are shown only when its ASVs are detected in both maternal and infant samples.

To further validate the transmission of gut bacteria from mothers to infants, we observed the species-level phylogenetic relationships for all 16 families and showed the phylogenetic trees of high-abundance genera in three representative families (Families 6, 9, and 15), which cover different enterotype patterns (Family 6, *Blautia*-Mix-*Bifidobacterium*; Family 9, Mix-Mix-Mix; Family 15, *Blautia-Staphylococcus-Bifidobacterium*), feeding methods (Family 6, Artificial; Family 9, Mixed; and Family 15, Breast), and maternal-infant allergic phenotypes (Family 6, Y_Y; Family 9, N_N; and Family 15, Y_N) (Table 4; Fig. 4). The close phylogenetic relationships between maternal and infant strains within the same species, particularly evident in the clustering patterns of multiple genera across different developmental stages (maternal, m0, and m3). Specifically, sequences representing the same ASV exhibit closer phylogenetic relationships between mother and infant than between different ASVs within the mother. Apart from the three genera (*Blautia*, *Staphylococcus*, and *Bifidobacterium*) that dominate in abundance and were selected as representative enterotypes, ASVs in other genera, such as *Enterococcus*, also exhibit this vertical transmission. In addition to the three representative families, the ASVs of the above four genera also exhibit vertical transmission in almost all other families (supplemental figure: Fig. S1 to S5). These findings provide strong evidence for the vertical transmission of gut microbiota. This species-level analysis enhances our understanding of the specific bacterial strains involved in maternal-infant transmission and their potential influence on infant CMPA development.

**TABLE 4:** Distribution of enterotypes and feeding patterns across maternal-infant pairs with different allergy phenotypes^*a*^^,^^*b*^

Family number	Maternal	M0	M3	Feeding methods	Phenotype
Family 1	Mix	Mix	Mix	Breast	N_N
Family 2	Mix	*Staphylococcus*	*Bifidobacterium*	Mixed	Y_Y
Family 3	*Blautia*	Mix	*Bifidobacterium*	Breast	Y_N
Family 4	*Blautia*	Mix	*Bifidobacterium*	Breast	Y_N
Family 5	Mix	Mix	*Bifidobacterium*	Mixed	N_N
**Family 6**	*Blautia*	**Mix**	*Bifidobacterium*	**Artificial**	**Y_Y**
Family 7	*Blautia*	*Staphylococcus*	*Bifidobacterium*	Mixed	Y_Y
Family 8	Mix	Mix	*Bifidobacterium*	Mixed	Y_Y
**Family 9**	**Mix**	**Mix**	**Mix**	**Mixed**	**N_N**
Family 10	*Blautia*	Mix	*Bifidobacterium*	Artificial	Y_Y
Family 11	*Blautia*	*Staphylococcus*	Mix	Mixed	Y_N
Family 12	Mix	Mix	Mix	Breast	Y_N
Family 13	Mix	Mix	*Bifidobacterium*	Mixed	N_Y
Family 14	*Blautia*	Mix	*Bifidobacterium*	Mixed	Y_Y
**Family 15**	*Blautia*	*Staphylococcus*	*Bifidobacterium*	**Breast**	**Y_N**
Family 16	*Blautia*	Mix	*Bifidobacterium*	Mixed	Y_Y

^*a*^ The predominant genera defining each enterotype were determined based on the LDA effect size. If the relative abundance of the predominant genus >10% among all genera within a sample, the enterotype was classified as the predominant enterotype (*Blautia*, *Staphylococcus*, or *Bifidobacterium*); otherwise, it was defined as the Mix type.

^*b*^ Boldface indicate families 6, 9, and 15, which were depict in Fig. 4.

## DISCUSSION

In our study, alpha diversity of the gut microbiota did not show significant differences between allergic and non-allergic samples (Fig. 2A and E). This suggests that it is essential to further investigate differences in specific bacterial taxa rather than focusing solely on overall microbial diversity. *Bifidobacterium* was identified as a biomarker for 3-month-old infants (m3) but was not recognized as a differential biomarker between the allergy and non-allergy groups (Fig. 1D and 2D). Contrary to some extent to previous studies that *Bifidobacterium* has been found to be reduced in abundance in food allergic individuals (23), our observations of gut enterotypes and feeding patterns in 16 families revealed that several m3 individuals with “Mix” enterotypes instead of “*Bifidobacterium*” were actually non-allergic. However, given that *Bifidobacterium* is one of the most common probiotics (24), we believe that infant allergies are not caused by *Bifidobacterium*. Instead, in response to allergic conditions, the abundance of beneficial bacteria such as *Bifidobacterium* may increase compensatorily, potentially counteracting the negative health effects. We look forward to more detailed and in-depth evidence to determine whether the bacteria involved in these patterns, particularly *Bifidobacterium*, serve as protective adaptations or contribute to the allergic state.

Feeding practices emerged as crucial modulators of microbiota development and infant allergy outcomes. The protective effect of exclusive breastfeeding was evident, with all breastfed infants remaining allergic-free regardless of maternal status (Table 4). This result is consistent with previous research that the diverse microorganisms in breast milk seed the infant’s gut microbiota. Breast milk contains growth-promoting nutrients and bioactive components, such as human milk oligosaccharides (HMOs), lactoferrin, and immunoglobulins, which contribute to the development of the infant’s immune system (25). It has been shown that certain genomes of infant gut commensals, in particular those of bifidobacterial species, are genetically adapted to utilize specific glycans of this human secretory fluid (22).

Our functional analysis, based on 16S-predicted metabolic pathways, suggests potential mechanistic links between early microbiota and immune system development through metabolic programming. The observed enrichment of amino acid metabolism pathways, particularly those involving sulfur-containing amino acids, implies a possible window during which microbial metabolic activities may influence immune function. Cysteine and methionine metabolism could be particularly relevant, given their roles in glutathione synthesis, immune cell proliferation, and T cell development (26, 27). Although the m3 group included infants fed with breast milk, formula, or mixed feeding, the enrichment of these immune-relevant metabolic pathways may still reflect the contribution of breast milk components, such as HMOs and immunoglobulins. Additionally, high levels of breast milk immune factors (IgA and cytokines) have been associated with a reduced risk of food allergy in infants (28), and breastfeeding has been linked to early neonatal regulatory T-cell expansion and immune tolerance (29). Taking the role of IgA as an example, its deficiency has been shown to disrupt the balance of gut microorganisms and exacerbate systemic immune disorders (30). *In vitro* studies have shown that secretory IgA (sIgA) reduces allergen translocation across the gut epithelium and directly binds to common food allergens, lowering their immunogenicity (31, 32). Animal studies further suggest that IgA-deficient mice are more susceptible to food allergies, and passive transfer of sIgA can mitigate allergen-induced immune activation (33, 34). These findings highlight the potential role of breast milk sIgA in shaping early immune tolerance and suggest that its absence or dysregulation may contribute to allergic disease development.

HMOs are among the key bioactive components in maternal milk that shape the gut microbiota of breastfed infants. Beyond serving as prebiotics that promote the growth of beneficial bacteria, particularly *Bifidobacterium*, HMOs have been shown to exert immunomodulatory effects through multiple mechanisms, including direct interactions with epithelial and immune cells, pathogen inhibition via decoy receptor activity, and modulation of intestinal barrier function (35, 36). The enrichment of starch and sucrose metabolism pathways observed in the m3 group may reflect a subset of breastfed infants within the group who had active HMO utilization by their microbiota. The ability of infants to metabolize different HMOs strongly correlates with their fecal microbiota composition, influencing microbial colonization and immune maturation. Recent studies suggest that HMOs may play a crucial role in allergy prevention by supporting oral tolerance development. They enhance the production of short-chain fatty acids, which contribute to regulatory T-cell expansion and immune homeostasis (37). Furthermore, specific HMOs can bind to immune-related receptors, modulating neonatal immunity beyond the gut and potentially reducing the risk of allergic diseases (38). In our study, the concurrent enrichment of starch and sucrose metabolism pathways suggests that early microbial communities may influence immune programming not only through protein metabolism but also via HMO utilization patterns. These metabolic signatures could serve as early indicators of CMPA predisposition and highlight HMOs as potential targets for CMPA prevention strategies. Thus, these metabolic signatures may capture breastfeeding-related microbial functions, suggesting that a proportion of infants benefited from early exposure to HMOs and sIgA. Future research should focus on identifying the specific HMOs and microbial interactions that drive these immunological effects, further elucidating their role in shaping long-term immune responses.

In addition to gut microbiota composition, allergic diseases are influenced by multiple other factors, including host gene mutation (39) and epigenetic modifications (40). In recent years, an increasing number of studies have highlighted the role of epigenetics in food allergy development (41). Early-life environmental factors, such as microbial exposure and feeding practices, may induce allergic responses in newborns through mechanisms like DNA methylation (42). However, due to various limitations, this study did not include genetic and epigenetic data. Instead, we explored the association between maternal and infant allergic symptoms based solely on gut microbial composition using 16S rRNA sequencing. While this approach provides valuable insights into microbiome-related patterns, integrating epigenetic analysis in future studies could offer a more comprehensive understanding of the underlying mechanisms of CMPA development. Several limitations should be considered when interpreting our findings. This study primarily explores the transmission of maternal allergy history to infant allergic symptoms. While we included both eczema and CMPA samples as allergic phenotypes in our analysis, the primary focus remained on CMPA due to its nature as a food allergy, which is more closely associated with gut microbiota composition that may play a mediating role in allergic development. Our results indicate that while infant eczema and CMPA samples share some microbial similarities, they may also exhibit distinct microbial patterns (Fig. 2E and F). However, due to the impact of the COVID-19 pandemic, the sample size in this study was relatively small, particularly for certain allergic phenotype combinations, which may limit the statistical power to fully assess the differences between eczema and CMPA. Additionally, variations in environmental exposures and the lack of standardized allergy classifications could have introduced confounding factors. The use of 16S rRNA sequencing, while informative, also has limitations compared to more comprehensive methods like metagenomic sequencing. Moving forward, our goal is to expand the cohort and further investigate the maternal-infant transmission of gut microbiota, particularly through breastfeeding, and its role in the prevention and protection against CMPA.

## MATERIALS AND METHODS

### Study population and eligibility criteria

This prospective study enrolled 50 infants born between December 2020 and June 2021. For each infant, maternal allergy history and feeding method (exclusive breastfeeding or formula feeding) were meticulously documented. The gender distribution was comparable across study groups, ensuring demographic balance. All infants were delivered vaginally, providing a consistent mode of birth across the study population and controlling for potential variations in initial microbiota colonization. This study was approved by the Ethics Committee of Hangzhou City, Yuhang District Maternal and Child Healthcare Hospital (Supplemental file 1: approval number: LLSC-KYKT-2021-0006-A, approved on 26 January, 2021). Written informed consent was obtained from all participating mothers for both themselves and their infants before enrollment in the study. All procedures were conducted in accordance with the Declaration of Helsinki.

Neonates were eligible for inclusion if they met the following criteria: (i) Gestational age between 37 and 42 weeks; (ii) Birth weight ranging from 2,500 to 4,000 g; (iii) Healthy with no malformations; (iv) Postnatal Apgar score greater than 7; (v) Singleton birth; (vi) Exclusively breastfed, formula-fed, or mixed feeding of breast milk and formula milk after birth; (vii) At least one parent, grandparent, or maternal grandparent with a history of allergy; and (viii) No family history of genetic metabolic diseases.

Infants were excluded from the study if any of the following conditions were present: (i) Maternal complications during pregnancy (e.g., diabetes, viral hepatitis, and intestinal obstruction); (ii) Maternal history of special drug use, smoking, drug abuse, or alcohol abuse; (iii) Neonatal conditions such as asphyxia, hypoxic-ischemic encephalopathy, intracranial hemorrhage, intrauterine infection, neonatal infectious disease, or necrotizing enterocolitis; (iv) Genetic metabolic diseases or congenital malformations in the newborn; (v) Use of antibiotics or probiotics within 2 weeks prior to sample collection; (vi) Maternal mammary diseases (e.g., mastitis and breast cancer); and (vii) Participation in other clinical trials.

### Sample collection and diagnostic assessments

Sample collection and labeling were conducted with meticulous care. Heat-sensitive labels containing maternal and infant information were printed at nurse stations and affixed to stool collection tubes. Fecal samples were collected from newborns (meconium) and mothers within 0–48 h postpartum, with follow-up samples taken at 3 months. Strict hygiene protocols were followed, including hand washing and avoiding urine contamination. A minimum of 2 g of fecal matter was collected using sterile spoons, targeting the inner portion of the stool. Newborn samples were immediately transferred to the neonatal department, while 3-month samples were delivered to the pediatric triage within 1 h. All samples were promptly frozen at −80°C and transported to the laboratory using dry ice.

In addition to negative controls, we also collected samples from infants with eczema, which were categorized as part of the allergic-positive cohort for comparative analysis. Both eczema and CMPA infants exhibited skin-related symptoms such as rash, itching, or dermatitis. CMPA samples additionally exhibited gastrointestinal symptoms (e.g., diarrhea and recurrent bloody stools). All CMPA infants underwent an oral food challenge, during which their mothers avoided milk protein-containing foods for 2–4 weeks or replaced standard formula with an amino acid-based formula. A CMPA diagnosis was finally confirmed if allergic symptoms significantly improved during this elimination phase and reappeared upon reintroducing cow’s milk protein. Skin prick tests and serum-specific IgE (sIgE) levels were further used as supplementary diagnostic measures (43).

### DNA extraction and 16S rRNA sequencing

Fecal DNA was extracted from 500 mg samples using the FastPure Host Removal and Microbiome DNA Isolation Kit (Vazyme Biotech Co., Ltd, Nanjing), following the manufacturer’s instructions as implemented by Shanghai Majorbio Bio-pharm Technology Co., Ltd. The integrity of extracted nucleic acids was visually assessed through electrophoresis on a 1.0% agarose gel containing ethidium bromide. DNA concentration and purity were determined using a Qubit dsDNA HS Assay Kit (Life Technologies, Carlsbad, CA, USA) and quantified with Ultra-micro spectrophotometer nano-600. All samples yielded DNA concentrations exceeding 100 ng/µL, meeting the requisite threshold for sequencing.

The V3-V4 hypervariable region of the bacterial 16S rRNA gene was targeted for amplification using universal bacterial barcoded primers: 322F (5′-ACGGHCCARACTCCTACGGAA-3′) and 796R (5′-CTACCMGGGTATCTAATCCKG-3′). PCR amplification was conducted under the following thermal cycling conditions: initial denaturation at 95°C for 3 min, followed by 30 cycles of denaturation at 95°C for 30 s, annealing at 55°C for 30 s, and elongation at 72°C for 45 s, with a final extension step at 72°C for 10 min. The resulting amplicons were purified and pooled according to the MGIEasy Universal DNA Library Prep Set (1000005250) protocol to generate the final sequencing library. High-throughput sequencing was subsequently performed on the BGISEQ-2000 platform (Beijing, China) to produce 300 bp paired-end reads, enabling comprehensive analysis of the microbial community composition.

### Microbiome data processing, statistical analyses, and phylogenetic tree construction

Raw 16S rRNA sequencing data underwent bioinformatic analysis using QIIME2 (44) (amplicon-2023.2) for amplicon sequence variant (ASV) determination. The initial taxonomic classification was performed using QIIME2’s VSEARCH (45) consensus classifier against the SILVA (46) 138-99 reference database with parameters set to 90%cent identity and 80% query coverage.

Taxonomic composition and relative abundance analyses were performed and visualized using the matplotlib module in Python (version 3.10.12). Alpha diversity metrics, including Shannon diversity indices, were calculated to assess within-sample diversity. Beta diversity was evaluated through PCoA based on Bray-Curtis distances. To identify differentially abundant taxa between experimental groups, LEfSe (47) analysis was conducted with an LDA score threshold of 2.0 and a significance level of *α* = 0.05. Potential functional profiles of the microbial communities were predicted using PICRUSt2 (48). Statistical analysis and visualization of the predicted functional pathways were performed using STAMP2 (49).

For phylogenetic tree construction, ASV sequences and their corresponding taxonomic annotations were obtained from individual sequencing data. ASVs were analyzed in family units, comprising maternal samples and their corresponding infant samples at birth (m0) and 3 months (m3). ASVs were filtered to exclude those lacking complete taxonomic assignments or labeled as “uncultured” and further refined based on 100% sequence identity and distinguished using numerical suffixes (_1, _2, _3, etc.) for inter-timepoint tracking. Sequences were aligned using MAFFT (50). After masking highly variable positions, phylogenetic trees were constructed using FastTree (51) with the GTR model and midpoint-rooted within the QIIME2 pipeline.

## Data Availability

Original sequencing data of each sample are deposited in the National Microbiology Data Center (NMDC) with accession number NMDC40090203.

## SUPPLEMENTAL MATERIAL

Figure S1 to S5 spectrum.01162-25-s0001.DOCX The vertical transmission of microbiota in more families.

Table S1 spectrum.01162-25-s0002.xlsx Basic biological information of the sample, such as gender, allergic state, age, weight, length, and so on.

Table S2 spectrum.01162-25-s0003.docx Comparison of basic characteristics of infants.

Table S3 spectrum.01162-25-s0004.txt Relative abundances of representative bacterial genera of 16 family.

ASM does not own the copyrights to Supplemental Material that may be linked to, or accessed through, an article. The authors have granted ASM a non-exclusive, world-wide license to publish the Supplemental Material files. Please contact the corresponding author directly for reuse.
