# Supplementary material for: Maternal gut microbiota transmission and early-life colonization patterns influence infant CMPA risk
Source: Microbiol Spectr. 2025 Oct 20;13(12):e01162-25. doi: 10.1128/spectrum.01162-25 (PMC12671192; doi:10.1128/spectrum.01162-25)
Supplement: Figure S1 to S5 — The vertical transmission of microbiota in more families. [file spectrum.01162-25-s0001.docx]

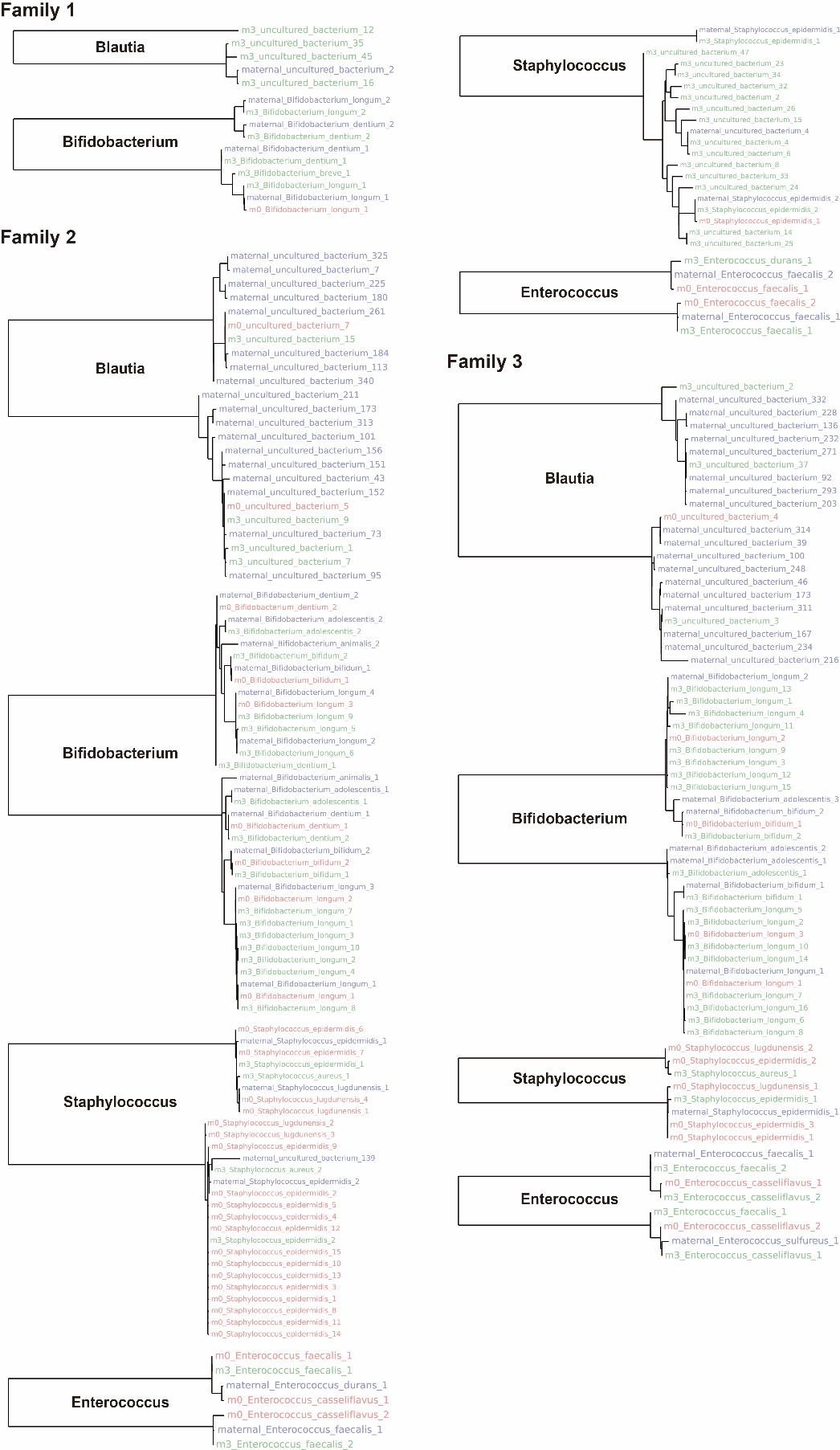


**Figure S1. Phylogenetic analysis of maternal-infant bacterial vertical transmission in Family1, 2 and 3.**

ASVs of the same species are distinguished by numerical suffixes. Leaf colors represent sample origin (purple, maternal; red, m0; green, m3). Phylogenetic trees of a genus within a family are shown only when its ASVs are detected in both maternal and infant samples.


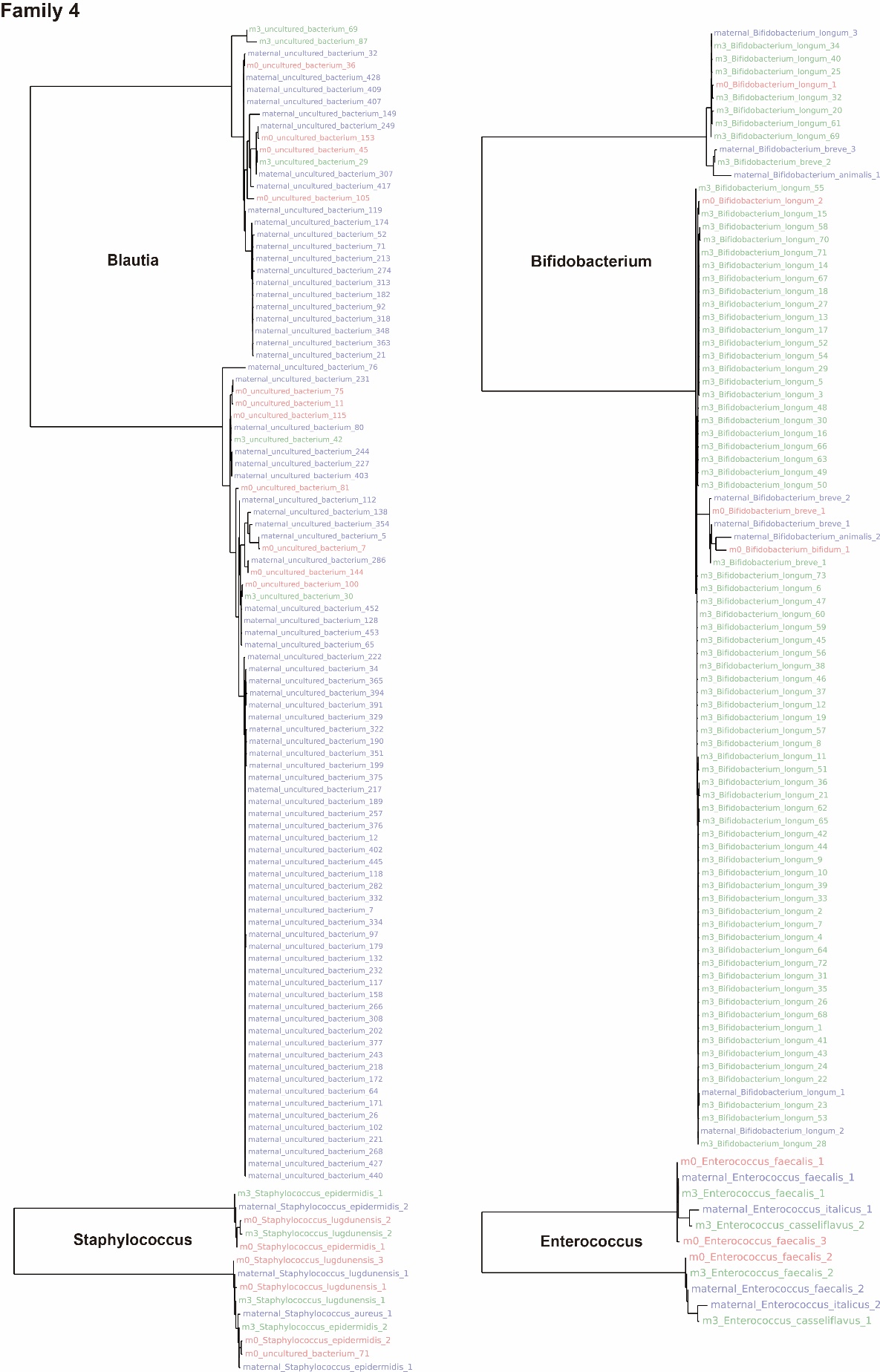


**Figure S2. Phylogenetic analysis of maternal-infant bacterial vertical transmission in Family4.**

ASVs of the same species are distinguished by numerical suffixes. Leaf colors represent sample origin (purple, maternal; red, m0; green, m3). Phylogenetic trees of a genus within a family are shown only when its ASVs are detected in both maternal and infant samples.


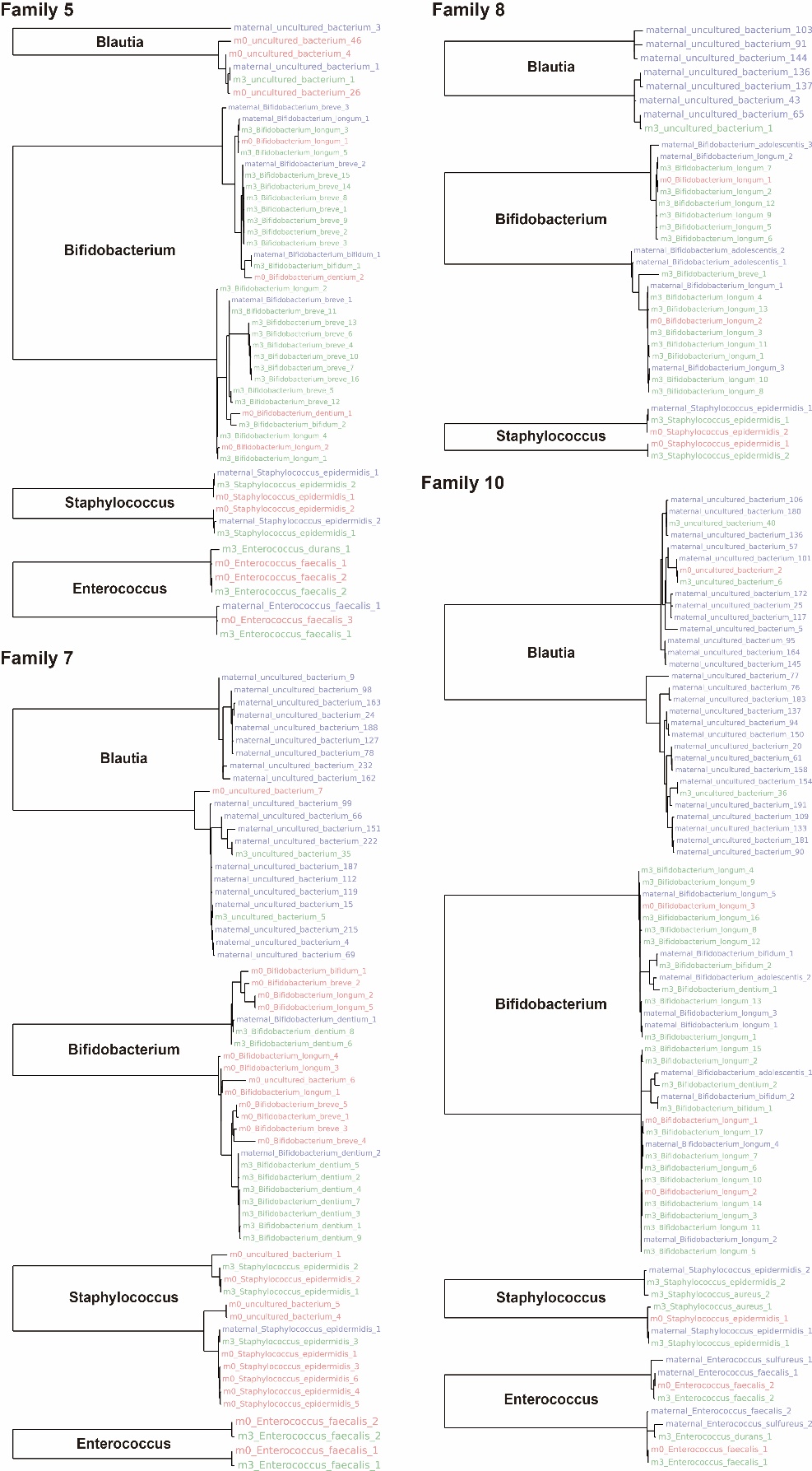


**Figure S3. Phylogenetic analysis of maternal-infant bacterial vertical transmission in Family5, 7, 8, and 10.**

ASVs of the same species are distinguished by numerical suffixes. Leaf colors represent sample origin (purple, maternal; red, m0; green, m3). Phylogenetic trees of a genus within a family are shown only when its ASVs are detected in both maternal and infant samples.


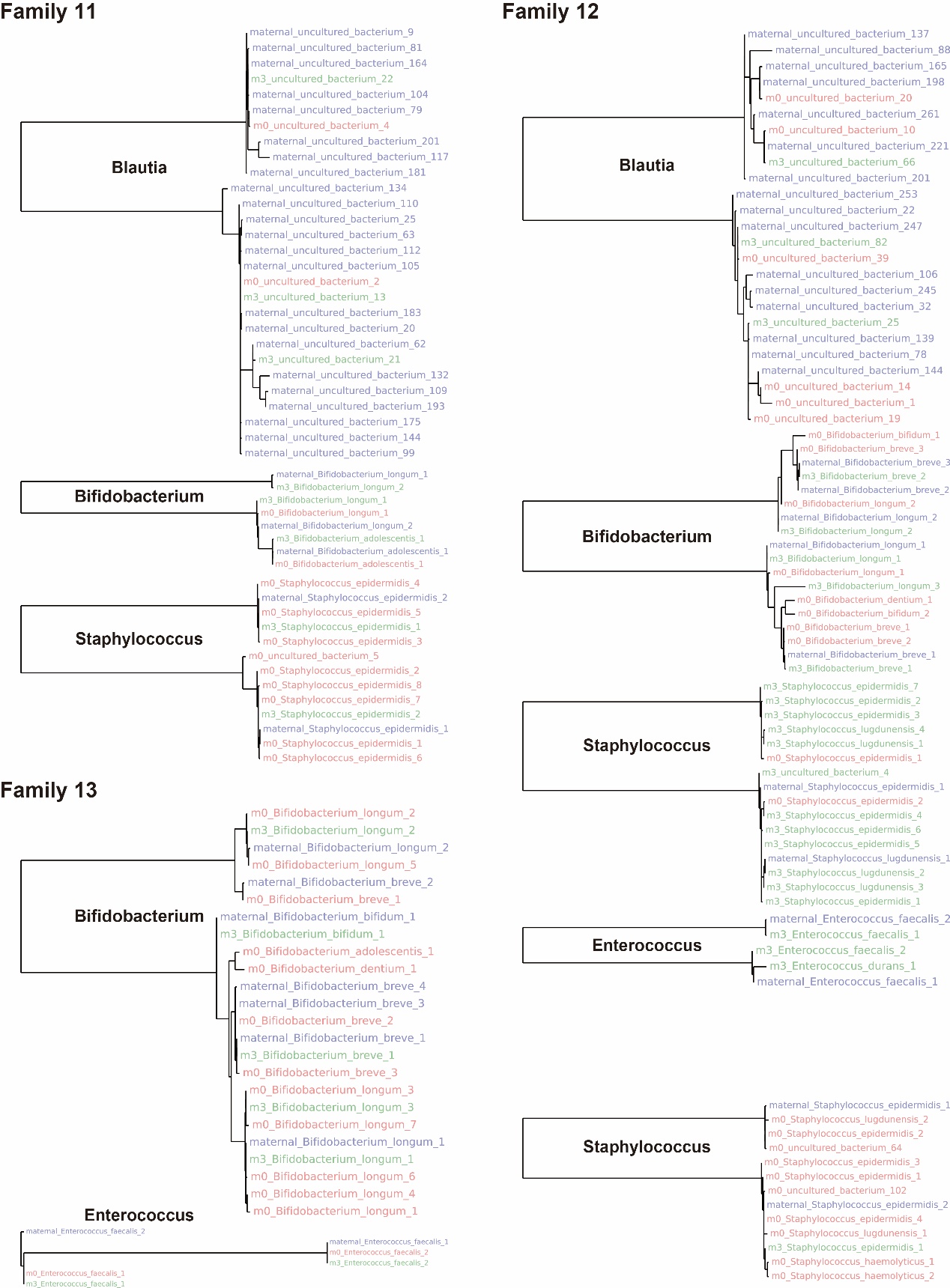


**Figure S4. Phylogenetic analysis of maternal-infant bacterial vertical transmission in Family11, 12, and 13.**

ASVs of the same species are distinguished by numerical suffixes. Leaf colors represent sample origin (purple, maternal; red, m0; green, m3). Phylogenetic trees of a genus within a family are shown only when its ASVs are detected in both maternal and infant samples.


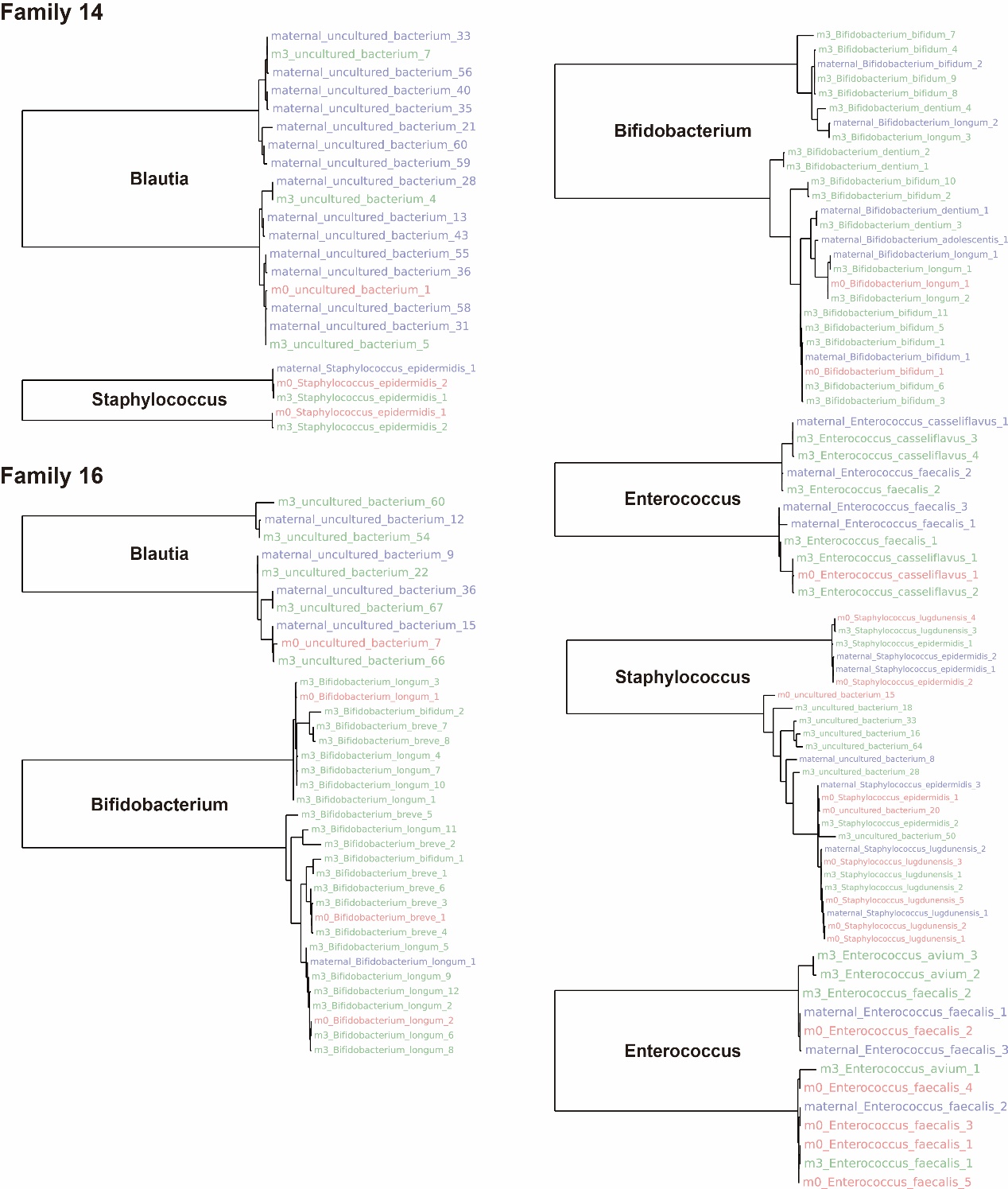


**Figure S5. Phylogenetic analysis of maternal-infant bacterial vertical transmission in Family14 and 16.**

ASVs of the same species are distinguished by numerical suffixes. Leaf colors represent sample origin (purple, maternal; red, m0; green, m3). Phylogenetic trees of a genus within a family are shown only when its ASVs are detected in both maternal and infant samples.
