# Supplementary material for: Maternal gut microbiota transmission and early-life colonization patterns influence infant CMPA risk
Source: Microbiol Spectr. 2025 Oct 20;13(12):e01162-25. doi: 10.1128/spectrum.01162-25 (PMC12671192; doi:10.1128/spectrum.01162-25)
Supplement: Table S2 — Comparison of basic characteristics of infants. [file spectrum.01162-25-s0003.docx]

Table S2. Comparison of basic characteristics of infants (kruskal-wallis test):

New-born baby (m0):

|  | Y_Y (n=15) | Y_N (n=19) | N_N (n=15) | *p*-value |
| --- | --- | --- | --- | --- |
| Length (cm) | 48-51 | 49-52 | 49-53 | 0.104 |
| Weight (kg) | 2.79-3.54 | 2.86-3.68 | 2.82-3.84 | 0.5699 |

Three-month-old infants (m3):

|  | Y_Y (n=10) | Y_N (n=6) | N_N (n=4) | *p*-value |
| --- | --- | --- | --- | --- |
| Length (cm) | 61-64 | 61-64 | 62-64 | 0.07705 |
| Weight (kg) | 6.2-7.5 | 6.85-7.25 | 3.6-7.2 | 0.9297 |
